# CXCL10 Is Associated with Increased Cerebrospinal Fluid Immune Cell Infiltration and Disease Duration in Multiple Sclerosis

**DOI:** 10.3390/biom13081204

**Published:** 2023-08-01

**Authors:** Stephanie N. Blandford, Neva J. Fudge, Craig S. Moore

**Affiliations:** 1 Faculty of Medicine, Division of Biomedical Sciences, Memorial University of Newfoundland, St. John’s, NL A1B 3V6, Canada; 2 Health Sciences Centre, Room HSC4364, 300 Prince Philip Drive, St. John’s, NL A1B 3V6, Canada

**Keywords:** multiple sclerosis, cerebrospinal fluid biomarker, CXCL10, immune cell trafficking, immune cell profiling

## Abstract

Background: Cerebrospinal fluid (CSF) is an important sampling site for putative biomarkers and contains immune cells. CXCL10 is a multiple sclerosis (MS)-relevant chemokine that is present in the injured central nervous system and recruits CXCR3+ immune cells toward injured tissues. Objective: Perform a comprehensive evaluation to determine a potential relationship between CXCL10 and various immune cell subsets in the CNS of MS and control cases. Methods: In MS and control cases, CXCL10 was measured in the CSF and plasma by ELISA. Immune cells within both the CSF and peripheral blood were quantified by flow cytometry. Results: Compared to non-inflammatory neurological disease (NIND) cases, MS cases had significantly higher CXCL10 in CSF (*p* = 0.021); CXCL10 was also correlated with total cell numbers in CSF (*p* = 0.04) and T cell infiltrates (CD3+, *p* = 0.01; CD4+, *p* = 0.01; CD8+, *p* = 0.02); expression of CXCR3 on peripheral immune cell subsets was not associated with CSF CXCL10. Conclusions: Elevated levels of CXCL10 in the CSF of MS cases are associated with increased T cells but appear to be independent of peripheral CXCR3 expression. These results support the importance of elevated CXCL10 in MS and suggest the presence of an alternative mechanism of CXCL10 outside of solely influencing immune cell trafficking.

## 1. Introduction

Multiple sclerosis (MS) is a chronic disease characterized by an inflammatory insult to the central nervous system (CNS) that results in demyelination and neurodegeneration. The cerebrospinal fluid (CSF) plays an immunologically important role within the CNS [1]. Peripherally born immune cells circulate throughout the CSF and contribute to the homeostatic immunological surveillance of the CNS [2,3]. In MS, previous studies have documented a shift in the diversity of cell types present within the CSF compared to controls, suggesting that the CSF may provide a source of novel biomarkers to be explored in attempts to better understand, diagnose, and prognosticate MS [4,5,6].

Cytokines and chemokines are critical for the induction, maintenance, and resolution of inflammatory processes throughout the body. In individuals with inflammatory conditions, like MS, cytokine and chemokine profiles are often reflected by an increased pro-inflammatory and decreased anti-inflammatory molecular profile. CSF has been previously investigated to identify the repertoire of cytokines and chemokines present in the CNS in MS. Profiling studies have revealed significant increases in several cytokines in MS, many of which were related to Th1-mediated immune responses [7,8,9]. However, additional studies contradicted this notion, thus leaving a lack of consensus in the literature [10,11,12].

Cytokine and chemokine profiles in CSF are likely rich sources of putative biomarkers. However, exactly which marker (or combination of markers) is the most valuable remains elusive and depends on the type of biomarker being considered. CXCL10 is a chemokine produced in response to pro-inflammatory stimulation and is involved in the chemoattraction of macrophages, monocytes, and activated T and NK cells via binding to the CXCR3 receptor [13]. In MS, elevations in CXCL10 levels have been observed compared to controls [7,14,15,16,17,18,19]. Indeed, it has been noted in early reports that increases in CSF CXCL10 were associated with both the total number of cells circulating within the CSF and higher patient disability [7,19].

While CXCL10 has been recognized as being dysregulated in CNS inflammation and MS, a comprehensive evaluation of its associative levels and effects on cellular infiltration into the CNS compartment have yet to be investigated. Herein, we demonstrate an increase in CSF CXCL10 in MS compared to controls and that CXCL10 is associated with increased T cells within the CSF compartment but not with the expression of its receptor, CXCR3, in the periphery.

## 2. Materials and Methods

### 2.1. Patient and Demographic Information

All experiments involving living human participants and human samples were approved by the Newfoundland Health Research Ethics Board, which follows the declaration of Helsinki. Written informed consent was obtained from all participants prior to study initiation.

CSF and blood samples were obtained from patients recruited from the Health Sciences Center Neurology Clinic (St. John’s, NL, Canada) or Horizon Health (Saint John, NB, Canada). For CSF and cell analyses, all patients were DMT-naïve; 39/84 patients were treated with steroids at the time of clinical relapse (1–166 months prior to LP). For plasma analyses, RRMS patients were recruited through the Health Research Innovation Team in Multiple Sclerosis (HITMS), an MS patient registry and biorepository at Memorial University of Newfoundland, St. John’s NL, Canada. All clinical parameters were determined based on assessment by an MS neurologist. Clinical and demographic information for the various cohorts is presented in Table 1, Table 2 and Table 3.

### 2.2. Preparation of Human CSF Samples

CSF was drawn by lumbar puncture (LP), and 2.5 mL was centrifuged to collect cells for flow cytometry. The remaining sample was also centrifuged (300× *g*) to remove cells. Cell-free CSF was aliquoted, frozen at −80 °C, and transferred to liquid nitrogen. Cells not used for flow cytometry were lysed in QIAzol^®^ (Qiagen, Germantown, MD, USA) and stored at −80 °C for RNA isolation and qPCR analysis.

### 2.3. Preparation of Human Blood Samples

Plasma was obtained by centrifugation of whole blood samples (300× *g*). Peripheral blood mononuclear cells (PBMCs) were isolated as described previously [20]. Blood was collected into EDTA-coated tubes (BD Vacutainer^®^). PBMCs were isolated from whole blood using Ficoll density gradient centrifugation (Thermo Fisher, Waltham, MA, USA) and SepMate™ Tubes (StemCell Technologies, Vancouver, BC, Canada). Once isolated, PBMCs were cryopreserved and stored in liquid nitrogen.

### 2.4. Cytokine Measurements

Assays were performed according to the manufacturer’s instructions. CSF levels of 27 cytokines, including CXCL10, were measured by Bio-Plex Pro™ Human Cytokine 27-plex Assay and analyzed on a Bio-Plex^®^ 200 system. Cytokine measurements in CSF and plasma were obtained for human CXCL10 by ELISA (R&D Systems, Minneapolis, MN, USA).

### 2.5. CSF Cell Flow Cytometry

CSF cells were quantified by flow cytometry in a subset of patients (Table 2). Cells were pelleted by centrifugation (300× *g*), resuspended in 100 uL flow buffer (PBS containing 1% bovine serum albumin, 2 mM EDTA, and 2 mM sodium azide), applied to a DURAclone IM Phenotyping BASIC tube (Beckman Coulter, Brea, CA, USA), mixed, and incubated at 4 °C for 30 min. The cells were washed in flow buffer and fixed with 100 µL 2% paraformaldehyde. Data were acquired using the CytoFLEX (Beckman Coulter, Brea, CA, USA), and all cells in the sample were quantified.

### 2.6. PBMC Flow Cytometry

Peripheral PBMCs were quantified from 10 healthy controls and 20 RRMS cases (Table 3). Of the RRMS cases, 10 had high CSF CXCL10, and 10 had low CSF CXCL10 (cut-off value of 569 pg/mL). PBMCs were thawed from cryopreservation and stained with LIVE/DEAD™ aqua (Invitrogen/Thermo, Waltham, MA, USA) in PBS and incubated for 30 min at 4 °C in the dark. The cells were washed and resuspended in flow buffer and added to an antibody cocktail (BD Biosciences: anti-CD3-PerCP, anti-CD14-APC, and anti-CXCR3-PE; BioLegend: anti-CD4-FITC, anti-CD8-APC/Cy7, and anti-CD19-PacBlue) and incubated at 4 °C for 30 min in the dark. Anti-IgG1k-PE (BD Biosciences, San Jose, CA, USA) was used as an isotype control for anti-CXCR3. The cells were washed and fixed with 2% paraformaldehyde, and data from 100,000 cells were acquired using the CyoFLEX (Beckman Coulter, Brea, CA, USA).

### 2.7. RNA Isolation and qPCR Analyses

Total RNA was isolated using the RNeasy^®^ Micro Kit with DNAse treatment (Qiagen, Hilden, Germany) and quantified by NanoDrop^SM^ 1000 spectrophotometer (Fischer Scientific, Waltham, MA, USA). A total of 200 ng RNA was reverse transcribed using M-MLV reverse transcriptase kit (Invitrogen, Burlington, CA, USA). Gene expression assays were performed using TaqMan Fast Universal PCR Master Mix (Applied Biosystems, Waltham, MA, USA) and TaqMan probes and primers for *cxcr3* (Applied Biosystems, Waltham, MA, USA).

### 2.8. Analysis

Statistical analyses were conducted using Prism 9 (GraphPad Software Inc, Boston, MA, USA). Flow cytometry data were analyzed using FlowJo™ v10.0 software (FlowJo, LLC, Ashland, OR, USA). Data are presented as mean ± SEM unless otherwise indicated, and *p* ≤ 0.05 was considered significant. In all analyses, the data were assessed for normality by the Shapiro–Wilk test, and nonparametric tests were performed if the data were not normally distributed.

Bio-Plex and ELISA data were analyzed by t-tests comparing RRMS and NIND cases and included a correction for multiple comparisons using the Holm–Sidak method when necessary. For Bio-Plex, values measured using extrapolation below the lowest standard were included in the analyses. The percentage of extrapolated values for each cytokine is documented in Table 4. No CXCL10 measurements were extrapolated. CSF CXCL10 measured by ELISA and Bio-Plex™ in matching samples were analyzed by simple linear regression and Pearson correlation test. CXCL10 in CSF and/or plasma and CSF NfL were compared to demographic variables using *t*-tests (sex), simple linear regression, Pearson’s correlation test (disease duration, age, time since last relapse) or Spearman’s correlation (EDSS), and one way ANOVA (DMT use).

Flow cytometry data were gated independently for each sample. For CSF flow cytometry, cell counts were correlated with CXF CXCL10 concentration using Spearman’s correlation test. CXCR3 expression in PBMC populations was analyzed between HC and RRMS groups using one-way ANOVA and was correlated to CSF CXCL10 using Spearman’s correlation test. qPCR for CXCR3 in cells isolated from CSF was displayed as 1/CT and correlated to CXCL10 levels by Spearman’s correlation test.

## 3. Results

### 3.1. BioPlex Analysis of CSF Revealed Increased Expression of CXCL10 in RRMS

**Figure 1:**
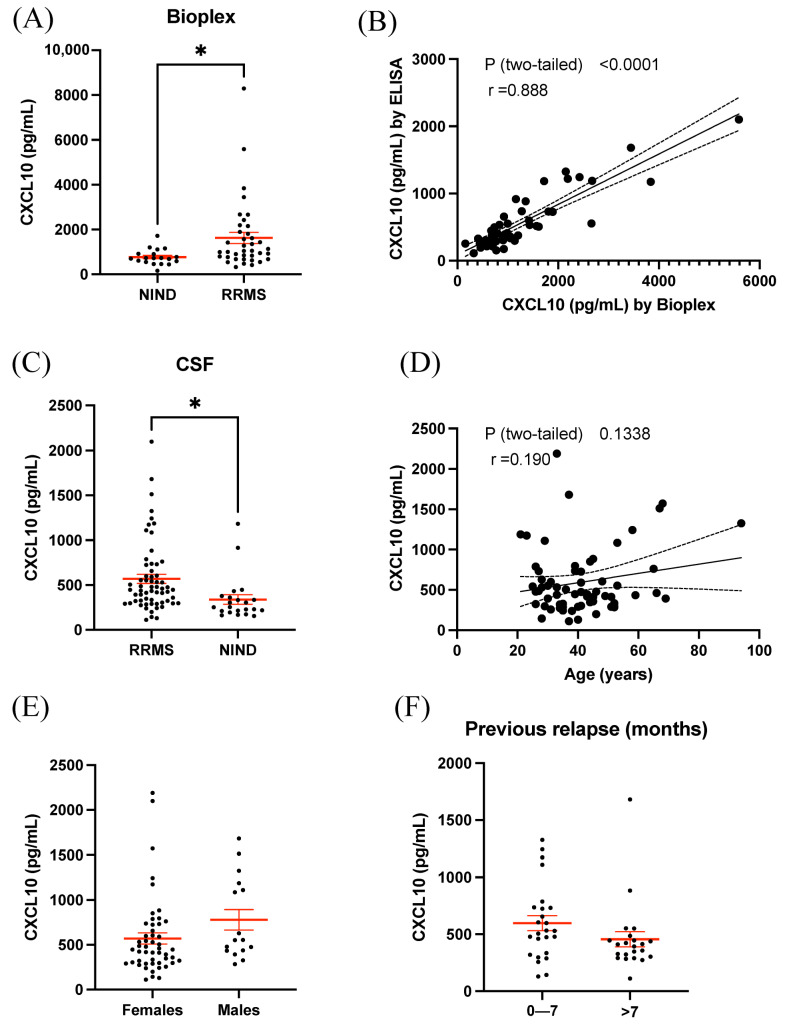
CXCL10 is elevated in RRMS CSF compared to NIND. (**A**) CSF CXCL10 is significantly elevated in RRMS cases compared to NIND controls when measured by BioPlex™. (**B**) CXCL10 concentrations measured by BioPlex™ and ELISA are significantly correlated. (**C**) ELISA measurements confirmed that CXCL10 is elevated in RRMS cases compared to NIND controls in a larger cohort. (**D**) CXCL10 was not correlated with age, nor were levels different between males and females (**E**). (**F**) Levels of CXCL10 were also comparable between those 0–7 months past their most recent previous relapse and those >7 months. Error bars represent mean ± SEM; * *p* < 0.05.

Initially, using Bioplex analysis, we wished to obtain an unbiased immunological profile of cytokines and growth factor expression within the CSF from RRMS patients and non-inflammatory neurological disease controls (NINDs; Table 1). In total, levels of 27 cytokines were measured (Table 4). Following a correction for multiple comparisons that included cytokine levels whereby a minimum of 80% of measurements were obtained via Bioplex (23 of the 27 cytokines measured in total), we observed a significant increase in CSF levels of CXCL10 in RRMS (1628 ± 70.32 pg/mL) compared to NIND (771 ± 80.2 pg/mL, *p* = 0.049, Figure 1A). Following the test for multiple comparisons, no other significant differences were measured between the RRMS and NIND groups for any remaining molecules that were measured.

### 3.2. ELISA Results Validated Elevated CXCL10 Expression in CSF of RRMS

Moving forward, all subsequent measurements of CXCL10 within CSF of RRMS and NIND cases were performed using an ELISA. A validation of the CXC10 results was therefore performed in a larger cohort (while also incorporating samples measured via Bioplex) using an ELISA (Table 2). Compared to the BioPlex assay, levels of CXCL10 were approximately 40% of the levels when measured by ELISA; however, when identical sample values were measured and compared between the two assays, CXCL10 levels were highly correlated (r = 0.888, *p* < 0.0001; Figure 1B). As such, using an ELISA, CSF CXCL10 levels were also significantly increased in RRMS (569 ± 49.9 pg/mL) compared to NIND (339 ± 53.0 pg/mL, *p* = 0.012, Figure 1C). In order to examine the possible influence of demographic variables on CXCL10 levels, it was determined that neither age (Figure 1D), sex (Figure 1E), or time since most recent MS relapse (Figure 1F) had a significant impact on CXCL10 levels.

### 3.3. Plasma CXCL10 Is Not Increased in RRMS Compared to Controls but Is Associated with Overall Disease Duration

**Figure 2:**
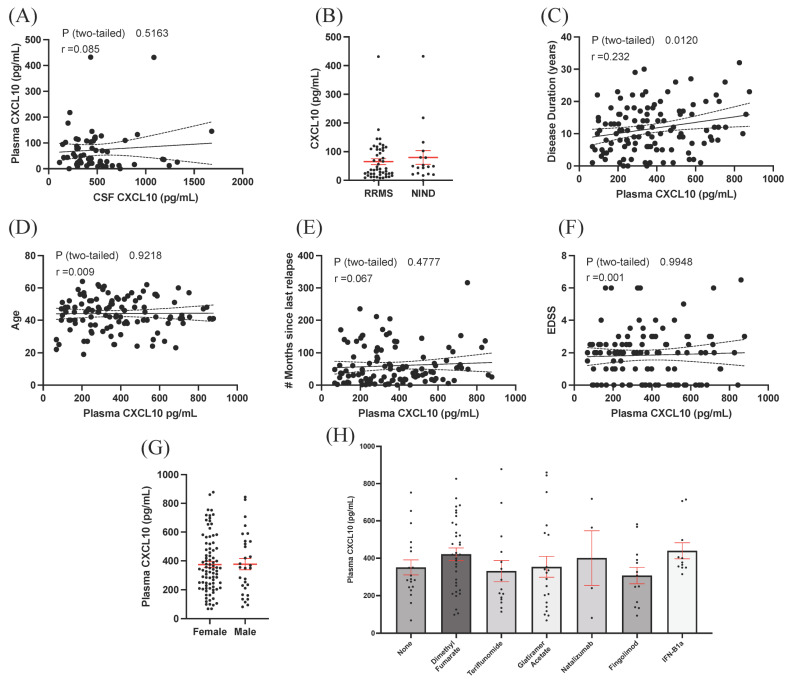
Analysis of CXCL10 in plasma. (**A**) Levels of CXCL10 in the plasma and CSF are not correlated with one another. (**B**) Levels of CXCL10 in plasma are no different between RRMS and NIND cases. (**C**) Plasma CXCL10 is significantly correlated with disease duration reported in years but not with patient age (**D**) number of months since most recent clinical relapse (**E**) or disability as measured by EDSS score (**F**). (**G**) Plasma CXCL10 levels were no different between males and females and were not associated with the use of any DMT (**H**). Error bars represent mean ± SEM.

To determine whether the increase in CXCL10 levels within the CSF of RRMS could also be detected in the more easily accessible blood plasma, we performed a correlational analysis of CSF CXCL10 levels vs. plasma CXCL10 levels within the same subjects. Interestingly, CSF and plasma CXCL10 were not correlated within the same subjects (Figure 2A), thus suggesting that plasma levels of CXCL10 cannot predict levels within the CSF. Furthermore, when comparing plasma levels of CXCL10, no differences were observed between the RRMS (65.4 ± 10.1 pg/mL) and NIND (79.8 ± 24.0 pg/mL, *p* = 0.515, Figure 2B) cohorts. Having determined that no differences in CXCL10 existed between the RRMS and NIND control groups, we examined a significantly larger cohort of RRMS cases (Table 3) to investigate whether plasma CXCL10 was significantly associated with various clinical or demographic variables. Using this dataset, we determined that plasma CXCL10 was significantly correlated with disease duration (r = 0.232, *p* = 0.012, Figure 2C), but there was no relationship with patient age (r = 0.009, *p* = 0.922, Figure 2D), number of months since previous relapse (r = 0.066, *p* = 0.478, Figure 2E), and disability as measured by EDSS (r = 0.0006, *p* = 0.995, Figure 2F). There was also no association between plasma CXCL10 and patient sex (males: 377 ± 39.1 vs. females 374 ± 21.2, *p* = 0.951, Figure 2G) or DMT use (F = 1.081, *p* = 0.380, Figure 2H).

### 3.4. Elevated CSF CXCL10 Is Associated with Increased T Cell Presence within the CSF

**Figure 3:**
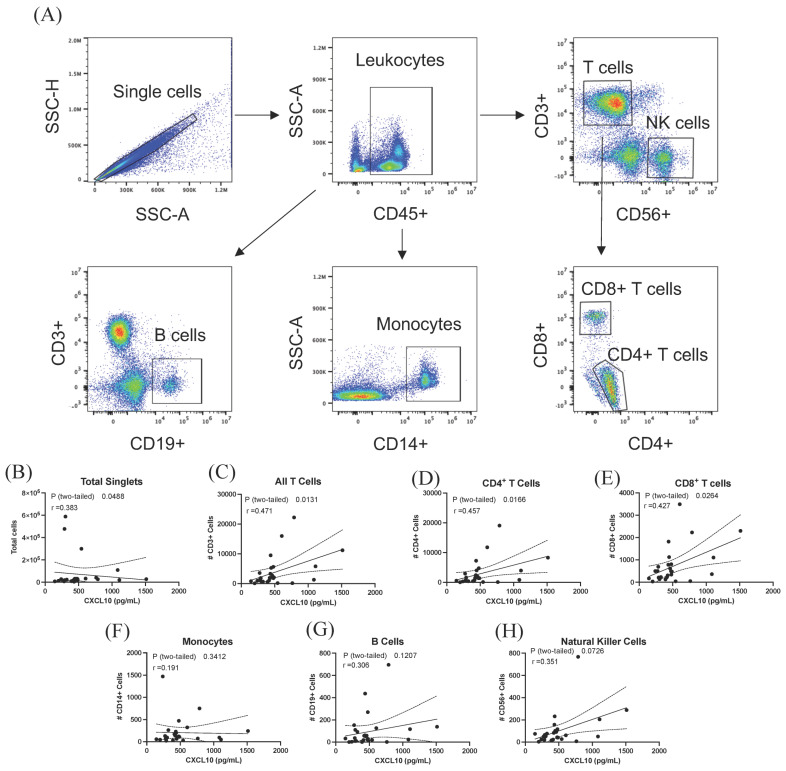
CXCL10 is correlated with T cells in the CSF. (**A**) Gating strategy to identify populations of immune cell subsets in the CSF. No correlations were observed with total singlets (**B**). Quantification of numbers of different immune cells in the CSF were correlated with CSF CXCL10 concentration in total CD3+ T cells (**C**), CD4+ T cells (**D**), and CD8+ T cells (**E**) but not with CD14+ monocytes (**F**), CD19+ B cells (**G**), or CD56+ natural killer cells (**H**).

Following centrifugation, using immune cells isolated from the CSF of documented RRMS cases, flow cytometry was used to phenotype the cells within the CSF; the gating strategy used is displayed in Figure 3A. The absolute numbers of each cell population were then correlated with CSF levels of CXCL10. CSF CXCL10 was significantly correlated with total numbers of T cells (r = 0.471, *p* = 0.013, Figure 3C), CD4+ T cells (r = 0.457, *p* = 0.017, Figure 3D), and CD8+ T cells (r = 0.427, *p* = 0.026, Figure 3E) but not with monocytes (r = 0.191, *p* = 0.341, Figure 3F), B cells (r = 0.306, *p* = 0.121, Figure 3G), or NK cells (r = 0.351, *p* = 0.071, Figure 3H).

### 3.5. Peripheral Expression of CXCR3 Does Not Differ between HC and RRMS and Is Not Associated with CSF CXCL10 Levels

**Figure 4:**
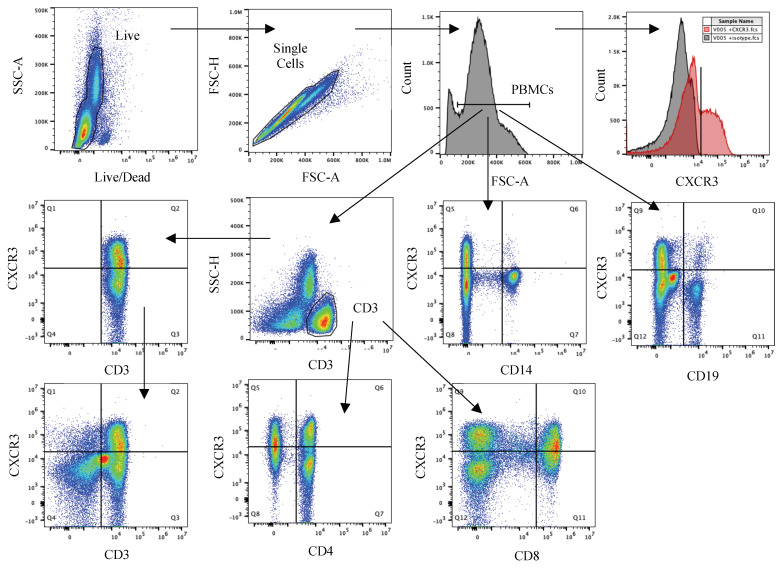
Peripheral PBMC gating strategy. Live cells were selected based on staining with a Live/Dead stain, then single cells based on FSC grouping. PBMCs were gated from the background using FSC-A. From the PBMC population, CXCR3 positive staining was identified using a cut-off based on comparing antibody staining (red) to its isotype (grey). CD3+ cells were selected from the PBMC population by gating using CD3 and SSC—H, and then split into CD4+ and CD8+ subpopulations. CD14+ monocytes and CD19+ B cells were gated from the whole PBMC populations using positive staining.

**Figure 5:**
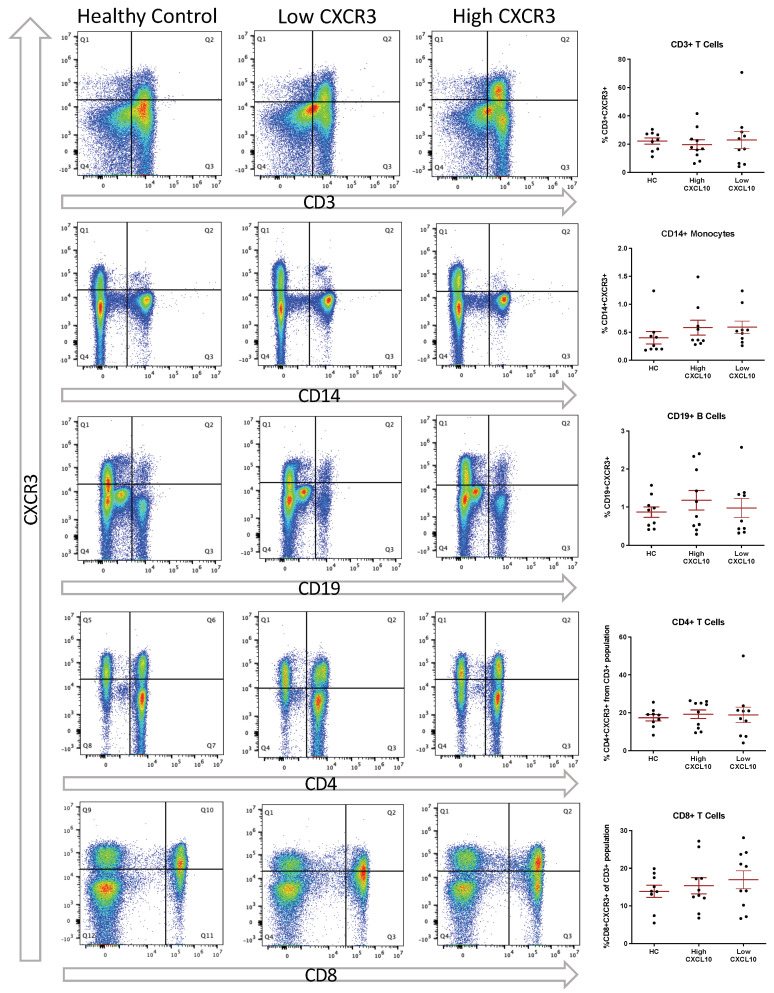
CSF CXCL10 is not associated with peripheral expression of CXCR3. Representative flow plots and quantifications reveal no difference in CXCR3 expression in CD3+ T cells, CD14+ monocytes, CD19+ B cells, CD4+ T cells, or CD8+ T cells between healthy controls and RRMS cases with high or low CSF CXCL10 concentrations. Error bars represent mean ± SEM.

Following analysis of CSF cells, flow cytometry was conducted on previously cryopreserved PBMCs from both healthy controls and RRMS cases. These experiments were conducted in order to measure levels of CXCR3+ (the cognate receptor for CXCL10) immune cells within circulation and to determine whether these cells were impacted by CSF levels of CXCL10. Expression of CXCR3 was assessed based on the gating strategy outlined in Figure 4. Cut-off values for positive CXCR3 expression were determined individually for each sample. Quantification of double-positive staining revealed no difference in CXCR3 expression between HC and RRMS groups in any of the populations tested. Of the whole PBMC population, 22.2 ± 2.22% were double-positive for CD3 and CXCR3 in HC, compared to 19.7 ± 3.43% and 23.0 ± 6.16% in the RRMS groups (high CSF CXCL10 (upper 50%) and low CSF CXCL10 (lower 50%), respectively; Figure 5; *p* = 0.805). Additionally, 0.402 ± 0.112% of PBMCs were double-positive for CD14 and CXCR3 in the HC group compared to 0.582 ± 0.133% (high CSF CXCR3) and 0.591 ± 0.109% (low CSF CXCR3) in the RRMS groups (Figure 5, *p* = 0.128). Finally, 0.863 ± 0.138% were double-positive for CD19 and CXCR3, compared to 1.18 ± 0.257% and 0.971 ± 0.249% in the RRMS groups (high CSF CXCL10 and low CSF CXCL10, respectively; Figure 5; *p* = 0.818).

**Figure 6:**
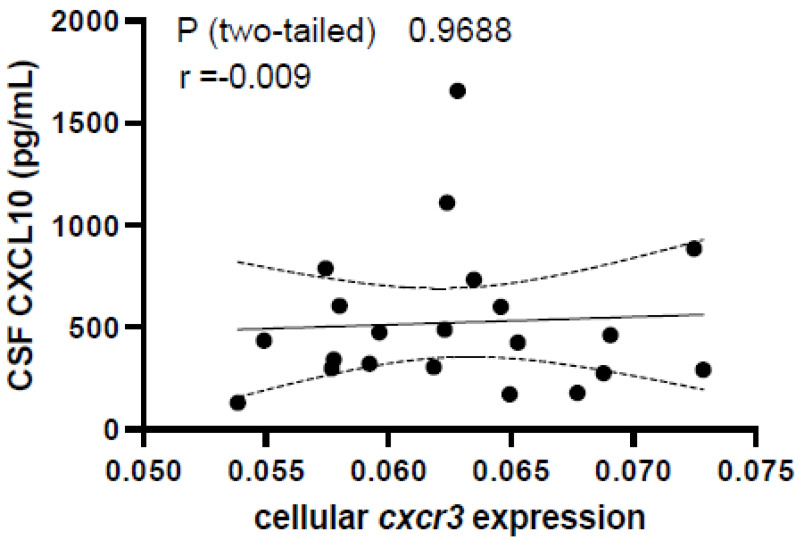
CSF CXCL10 is not correlated with *cxcr3* expression in pooled cells in CSF.

Of the CD3+ population, 17.4 ± 1.68% were double-positive for CD4 and CXCR3 in the HC group, compared to 19.2 ± 2.25% and 18.9 ± 4.07% in the RRMS groups (high CSF CXCL10 and low CSF CXCL10, respectively; Figure 5; *p* = 0.629). In addition, 13.9 ± 1.61% were double-positive for CD8 and CXCR3 in the HC group, whereas 15.4 ± 2.14% (high CSF CXCR3) and 17.0 ± 2.40% (low CSF CXCR3) were observed double-positive for CD8 and CXCR3 in the RRMS groups (Figure 5; *p* = 0.668). qPCR for CXCR3 was performed on cells collected from MS CSF. We observed that the expression of *cxcr3* mRNA in CSF cells was not correlated with CSF CXCL10 (Spearman’s r = −0.009, *p* = 0.969; Figure 6).

## 4. Discussion

The first goal of this study was to compare immunological profiles in human RRMS and NIND CSF samples, and investigate the pathological relevance to MS. We demonstrated that CXCL10 levels in the CSF were significantly elevated in RRMS compared to controls; levels were significantly correlated with numbers of T cells present in the CSF but were unrelated to the expression of the CXCL10 receptor, CXCR3, on immune cells in either the CSF or periphery.

Phenotyping CSF via a BioPlex™ immunoassay simultaneously measuring 27 cytokines/chemokines in each sample revealed little difference between RRMS and NIND CSF. We demonstrated that only CXCL10 was differentially regulated in RRMS compared to controls (Table 4). These results are in line with previous studies that have documented similar results [7,15,17,19,21]. A consensus of the literature suggests this finding may not be unique to MS but is rather reflective of an inflammatory CNS environment that occurs in MS and several other inflammatory neurological conditions/diseases [15,17,22].

We demonstrated that CXCL10 levels in the CSF were not associated with patient age (Figure 1D), sex (Figure 1E), or previous relapse activity (Figure 1F) and show that CXCL10 levels in the plasma vs. CSF were not correlated (Figure 2B). This suggests that plasma CXCL10 levels cannot be predictive of CSF levels; similar results have been previously reported [7,21]. Taken together, these results suggest that the blood compartment may not necessarily be reflective of the inflamed CNS compartment and further emphasizes the need to study CSF to help discover novel biomarkers in neuroinflammatory diseases.

We sought to determine whether plasma levels of CXCL10 were significantly associated with clinical and/or demographic variables in a cohort of 116 RRMS cases. CXCL10 was significantly correlated with overall disease duration (Figure 2C), but it was not associated with any other clinical or demographic variable investigated (Figure 2E–H). While several previous studies have compared CSF to blood levels of CXCL10, few have investigated any associations with clinical variables. One study compared clinical and demographic variables in serum CXCL10 in MS cases; the only significant correlation observed was between serum CXCL10 and age, but only in progressive MS cases [21].

Since CXCL10 is a known chemokine, we used flow cytometry to immunophenotype cells in the CSF of MS cases. When correlated to levels of CSF CXCL10, CXCL10 was significantly correlated with total cells in CSF and T cells (both CD4+ and CD8+; Figure 3). No other significant correlations were observed (Figure 3E–G). Previous studies investigating the association between CSF CXCL10 and immune cell infiltration have yielded conflicting results [17,19]. Consistent with our results, Sorensen et al. observed a significant correlation between the number of cells in the CSF and CXCL10 in the MS group but not in the NIND group [19]; another study noted no association [17]. The failure of these early studies to sub-categorize the cells into different populations may also underlie the discrepancies between these studies and the results presented here. To our knowledge, this is the first study to offer a comprehensive analysis of the relationships between CXCL10 and specific cell types in the CSF.

In parallel with these works, several other studies have used flow cytometry to quantify the expression of CXCR3 on peripheral and CSF T cells; these studies have also presented conflicting results [23,24,25]. One study documented an increase in CXCR3-expressing T cells in the blood and CSF in MS compared to NIND, driven by untreated patients since patients treated with IFN-β were no different from controls [23]. Interestingly, this study also found a higher proportion of CXCR3+ T cells in the CSF compared to the blood in matched samples, suggesting that the CXCR3/CXCL10 axis is an important driver of T cell infiltration into the CNS. However, no differences in the CSF CXCL10 levels were observed between MS and NIND cases [23]. A separate study published the same year reported similar results that the proportion of T cells expressing CXCR3 (CD4+ and CD8+ subsets) was higher in the CSF compared to blood. However, no difference between the MS and the control groups (NIND and healthy) was noted [24]. This study also reported that the percentage of CXCR3+ T cells in blood and CSF did not correlate with several clinical factors, including CSF CXCL10 concentration [24]. A third study documented a significantly higher percentage of CD4+CXCR3+ cells in the blood of active RRMS patients compared to stable, which may have implications for the above-mentioned studies, as neither separated their cohorts into active vs. non-active cases [25]. Nevertheless, this study also reported no correlation between CXCR3+ T cell populations in the blood or the CSF and CSF CXCL10 [25].

In the studies mentioned above, there was no consideration of the overall T cell population (both expressing and not expressing CXCR3) in relation to CXCL10 levels. This could explain why our results demonstrate a significant correlation between CSF CXCL10 and T cells in the CSF, while others did not. As the CSF flow cytometry performed in this study was a routine protocol performed as part of the general immunophenotyping of patient samples enrolled in our biorepository, flow cytometry for CXCR3 on CSF cells was not included, which is a limitation of our current study. We did perform qPCR for CXCR3 on pooled cells in the CSF samples and found that CXCR3 expression was not correlated with CSF CXCL10 (Figure 6), suggesting that CXCL10 is not related to the expression of *cxcr3* mRNA in leukocytes within the CSF. It is possible, however, that cells migrating based on a CXCL10/CXCR3 axis lose expression of CXCR3 once they have reached the high CXCL10 environment in the CNS. However, our data suggest that peripheral CXCR3 expression is not different between MS cases and controls and may potentially have no association with CSF CXCL10 (Figure 5). These data lead to the question of whether chemotaxis via the CXCR3/CXCL10 axis represents the sole function of CXCL10 in the CNS in MS cases. Several other routes of entry for leukocytes to enter the brain exist [26]. Therefore, the CXCL10/CXCR3 axis is not solely responsible for the infiltration of leukocytes and may represent the route of entry of only a minority of cells.

Along with CXCL10, the CXCR3 receptor also binds CXCL9 and CXCL11 [27]. Like CXCL10, increased CSF and serum CXCL9 have been documented in MS cases [28,29]. Serum CXCL9 has also recently been shown to correlate with clinical indicators such as lesion number and volume, both in the brain and spinal cord [28]. Highly specific spatial and temporal regulation of the CXCR3 ligands, combined with multiple splice variants with differing intracellular signaling pathways and affinity for each ligand, generates immense complexity in unravelling the specific roles in inflammation and autoimmunity [27]. Therefore, we cannot discount the role of CXCL9 and CXCL11 in the chemotaxis of immune cells into the CSF in MS. Measuring these chemokines was outside the scope of the current study but represents a key future direction.

Nevertheless, in summary, the present study provides evidence that CSF CXCL10 is elevated in RRMS cases compared to NIND and that it is not significantly associated with clinical or demographic variables. We also show that plasma CXCL10 is significantly correlated with disease duration but with no other clinical or demographic variables investigated. We provide evidence that CSF CXCL10 is significantly correlated with the number of T cells in the CSF, but this is unrelated to the expression of CXCR3 in the CSF or in the periphery. Taken together, these results support the importance of elevated CXCL10 in MS and other neurological disorders and support an alternative mechanism of CXCL10 outside of immune cell trafficking into the CNS.

## Figures and Tables

**Table 1:** CSF Bioplex Cohort (RRMS and NIND) and demographic information.

	RRMS (*n* = 37)	NIND (*n* = 19)
Age (years; mean ± SD)	39.3 ± 13.7	50.3 ± 17.4
F:M ratio	3.2:1	2.2:1
Most recent relapse (months; mean ± SD)	13.2 ± 18.7	N/A

NIND group includes patients with chronic fatigue (1), migraine (4), idiopathic intercranial hypertension (IIH) (12), spasticity (1), and non-inflammatory neurogenic bladder (1).

**Table 2:** CSF ELISA Cohort (RRMS and NIND) and demographic information.

	RRMS (*n* = 61)	NIND (*n* = 21)
Age (years; mean ± SD)	40.9 ± 13.6	46.0 ± 14.7
F:M ratio	2.6:1	4.2:1
Most recent relapse (months; mean ± SD)	18.2 ± 30.2	N/A

NIND group includes patients with chronic fatigue (1), migraine (4), idiopathic intercranial hypertension (IIH) (14), spasticity (1), and non-inflammatory neurogenic bladder (1).

**Table 3:** Plasma ELISA Cohort (RRMS expanded) and demographic information.

	RRMS (*n* = 116)
Age (years; mean ± SD)	44.1 ± 10.2
F:M ratio	2.9:1
Most recent relapse (months; mean ± SD)	60.0 ± 58.9
Disease duration (years; mean ± SD)	11.6 ± 7.36
Disease modifying therapy use	
None	16.4%
GA	17.2%
Natalizumab	3.45%
Fingolimod	11.2%
DMF	29.3%
Teriflunomide	12.9%
IFNβ-1a	9.48%

**Table 4:** Comparison of CSF cytokines measured by BioPlex between RRMS and NIND cases. *p*-values are adjusted for multiple comparisons. Cytokines that did not achieve at least an 80% detection rate were excluded from the statistical analysis.

Cytokine	% Detected		% Extrapolated		t Ratio	df	*p*-Value
IL-1β	RRMS	100%	RRMS	55.3%	1.454	20.51	0.970268
	NIND	100%	NIND	21.1%			
IL-1RA	RRMS	100%	RRMS	0%	0.6334	21.45	0.995249
	NIND	100%	NIND	0%			
IL-2	RRMS	100%	RRMS	78.9%	0.8841	24.84	0.995249
	NIND	100%	NIND	25.6%			
IL-4	RRMS	100%	RRMS	0%	1.099	24.20	0.992497
	NIND	100%	NIND	5.26%			
IL-5	RRMS	84.2%	RRMS	59.4%	0.5702	21.61	0.995249
	NIND	84.2%	NIND	50.0%			
IL-6	RRMS	100%	RRMS	13.2%	1.253	25.75	0.98893
	NIND	100%	NIND	0%			
IL-7	RRMS	100%	RRMS	2.63%	1.126	31.14	0.992497
	NIND	100%	NIND	5.26%			
IL-8	RRMS	100%	RRMS	0%	0.7736	22.40	0.995249
	NIND	100%	NIND	0%			
IL-9	RRMS	100%	RRMS	5.26%	0.8327	24.40	0.995249
	NIND	100%	NIND	5.26%			
IL-10	RRMS	92.1%	RRMS	20.0%	1.008	21.56	0.992497
	NIND	94.7%	NIND	16.7%			
IL-12p70	RRMS	97.4%	RRMS	32.4%	0.6928	23.25	0.995249
	NIND	100%	NIND	36.8%			
IL-13	RRMS	100%	RRMS	0%	0.06743	28.31	0.997161
	NIND	100%	NIND	0%			
IL-15	RRMS	47.4%	RRMS	0%	n/a	n/a	n/a
	NIND	42.1%	NIND	25.0%			
IL-17	RRMS	100%	RRMS	63.2%	1.145	24.37	0.992497
	NIND	100%	NIND	47.4%			
CXCL10	RRMS	100%	RRMS	0%	3.252	44.00	0.04945*
	NIND	100%	NIND	0%			
Eotaxin	RRMS	100%	RRMS	0%	0.3407	24.91	0.995249
	NIND	100%	NIND	0%			
FGF Basic	RRMS	97.4%	RRMS	100%	1.108	29.20	0.992497
	NIND	94.7%	NIND	88.9%			
G-CSF	RRMS	100%	RRMS	0%	1.359	22.04	0.980866
	NIND	100%	NIND	0%			
GM-CSF	RRMS	89.4%	RRMS	20.6%	0.3552	28.87	0.995249
	NIND	89.4%	NIND	11.8%			
IFN-γ	RRMS	73.7%	RRMS	10.7%	n/a	n/a	n/a
	NIND	78.9%	NIND	20.0%			
MCP-1	RRMS	100%	RRMS	0%	1.603	18.75	0.940469
	NIND	100%	NIND	0%			
MIP-1a	RRMS	100%	RRMS	0%	2.158	50.76	0.550753
	NIND	94.7%	NIND	0%			
PDGF-bb	RRMS	42.1%	RRMS	50.0%	n/a	n/a	n/a
	NIND	47.4%	NIND	22.2%			
MIP-1b	RRMS	100%	RRMS	0%	1.193	20.56	0.991874
	NIND	100%	NIND	0%			
RANTES	RRMS	100%	RRMS	26.3%	0.01761	34.10	0.997161
	NIND	100%	NIND	26.3%			
TNFα	RRMS	100%	RRMS	34.2%	0.736	33.74	0.995249
	NIND	100%	NIND	15.8%			
VEGF	RRMS	76.3%	RRMS	3.44%	n/a	n/a	n/a
	NIND	84.2%	NIND	6.25%			

## Data Availability

The data presented in this study are available on request from the corresponding author. The data are not publicly available due to ethical and privacy reasons.
